# Incidence and costs of injuries to children and adults in the United States

**DOI:** 10.1186/s40621-018-0167-6

**Published:** 2018-10-08

**Authors:** Mark R. Zonfrillo, Rebecca S. Spicer, Bruce A. Lawrence, Ted R. Miller

**Affiliations:** 1 Hasbro Children’s Hospital and Alpert Medical School of Brown University, Providence, RI USA; 2 Pacific Institute for Research and Evaluation, Calverton, MD USA; 3 Curtin University School of Public Health, Perth, Australia; 4 Department of Emergency Medicine, Alpert Medical School at Brown University, 55 Claverick St., 2nd floor, Providence, RI 02903 USA

## Abstract

**Background:**

Injuries are a leading cause of death and acquired disability, and result in significant medical spending. Prior estimates of injury-related cost have been limited by older data, for certain population, or specific mechanisms.

**Findings:**

This study estimated the incidence of hospital-treated nonfatal injuries in the United States (US) in 2013 and the related comprehensive costs. Injury-related emergency department (ED) visits and hospitalizations were identified using 2013 Healthcare Cost and Utilization Project (HCUP) data. Models estimated the costs of medical spending and lost future work due to injuries in 2013 U.S. dollars. A total of 31,038,072 nonfatal injury-related hospitalizations and ED visits were identified, representing 9.8 per 100 people. Hospital-treated nonfatal injuries cost an estimated $1.853 trillion, including $168 billion in medical spending, $223 billion in work losses, and $1.461 trillion in quality of life losses.

**Conclusions:**

Approximately one in 10 individuals in the US is treated in the hospital for injury each year, with high corresponding costs. These data support priority-setting to reduce the injury burden in the US.

## Background

Injuries are a leading cause of death and acquired disability in children and adults, and result in significant medical spending nationwide. Prior estimates of injury-related cost have been from much older data (Danseco et al. 2000; Miller et al. 2000; Corso et al. 2006), for certain populations (e.g., children (Miller et al. 2000; Zaloshnja et al. 2012; Roy et al. 2008), Medicaid recipients (Roy et al. 2008)), for certain mechanisms (e.g., consumer products (Lawrence and Miller, 2014), agricultural (Zaloshnja et al. 2012)), or for cost totals that only include medical care (Dieleman et al. 2016). This study provides contemporary national estimates of the incidence of hospital-treated nonfatal injuries for children and adults in the United States in 2013 and the related comprehensive costs by age, household income, payer, metropolitan residence, disposition, injury severity score, injury mechanism and injury intent. While incidence describes the magnitude of the problem, costs provide a better measure of burden by accounting for multiple injury consequences – e.g., severity, disability – in a single unit of measurement.

## Methods

Injury-related emergency department (ED) visits and hospitalizations were identified using 2013 Healthcare Cost and Utilization Project (HCUP) sample-based ED (Agency for Healthcare Research and Quality (AHRQ), n.d.) and inpatient (Agency for Healthcare Research and Quality (AHRQ)a, n.d.) datasets. This study used deidentified administrative data and was exempt from review by our institution. Injuries were defined as diagnoses 800–995 in the International Classification of Diseases, Ninth Revision, excluding late effects, 905–909. We used the standard external-cause matrix from the Centers for Disease Control and Prevention. (Centers for Disease Control and Prevention (CDC), n.d.-a; Centers for Disease Control and Prevention (CDC), n.d.-b).

We applied an established US injury cost model to the HCUP datasets to estimate the costs of injuries in 2013 U.S. dollars (Lawrence et al. 2015; Spicer et al. 2011). The Agency for Healthcare Research and Quality (AHRQ) weights HCUP Nationwide Emergency Department Sample (NEDS) and Nationwide Inpatient Sample (NIS) data to provide national estimates. 2014 NEDS estimates are based on sampling non-admitted cases from all EDs in 34 states. 2014 NIS estimates are based on a one-in-five random sample of inpatient discharges from every hospital in 44 states and the District of Columbia. (Agency for Healthcare Research and Quality (AHRQ), n.d. Agency for Healthcare Research and Quality (AHRQ)a, n.d.).

Costs include medical spending and lost future work. In addition, we take a societal perspective by including the estimated value of lost quality of life. Medicalspending includes hospital and professional services, emergency transport, rehabilitation, prescriptions, home health care, and medical equipment. Lost future work includes future value of work that patients would be unable to do if they were killed or permanently disabled. Future costs were stated in present value using a 3% discount rate. (Lawrence and Miller 2014). We valued quality of life lost with a widely published value of $152,733 per quality-adjusted life year, net of work loss (Miller and Hendrie, 2013). We used the same value per QALY of present-value life expectancy for all people. Because life-expectancy varies by age and sex, the total QALY loss to a death or permanently disabling injury also does.

## Results

### Incidence

A total of 31,038,072 nonfatal injury-related hospitalizations and ED visits were identified in 2013 (Table 1). This represents 9.8 per 100 people.

**Table 1:** Cost of Hospital-Treated Nonfatal Injuries in the United States, 2013 (2013 United States $)

	Number	Medical	Work Loss	Quality of Life	Total Cost	Total Cost per Injury
All	31,038,072	$167,968,253,878	$223,116,101,911	$1,461,482,900,000	$1,852,567,255,789	$59,687
Age Group						
< 1	261,308	$1,432,470,125	$1,789,973,631	$22,287,154,385	$25,509,598,141	$97,623
1–11	4,447,463	$12,032,484,055	$18,626,763,126	$181,322,230,105	$211,981,477,286	$47,663
12–17	2,823,241	$8,816,169,603	$13,443,289,763	$145,256,948,411	$167,516,407,777	$59,335
18–24	3,773,148	$13,600,427,953	$23,698,584,864	$186,399,030,940	$223,698,043,757	$59,287
25–34	4,442,848	$17,457,020,563	$36,742,723,349	$205,840,515,715	$260,040,259,627	$58,530
35–44	3,711,266	$15,005,365,402	$34,704,621,851	$150,463,641,515	$200,173,628,768	$53,937
45–64	6,458,720	$40,319,428,609	$65,703,484,682	$314,280,411,223	$420,303,324,514	$65,075
65+	4,800,513	$59,285,698,881	$28,387,430,398	$255,529,916,815	$343,203,046,094	$71,493
Household Income Quartile						
0-25th percentile	9,572,854	$47,498,738,599	$67,315,490,069	$419,419,736,702	$534,233,965,370	$55,807
26th–50th percentile	8,347,113	$43,448,674,708	$57,933,681,503	$380,949,318,832	$482,331,675,043	$57,784
31st-75th percentile	6,923,897	$39,294,070,461	$50,758,794,647	$339,582,090,153	$429,634,955,261	$62,051
76th–100th percentile	5,533,774	$33,659,639,931	$40,997,146,789	$284,762,942,717	$359,419,729,437	$64,950
Payer						
Medicare	5,599,892	$62,116,899,402	$40,517,276,172	$297,763,145,616	$400,397,321,190	$71,501
Medicaid	7,429,391	$29,171,568,219	$45,103,953,532	$314,054,136,094	$388,329,657,845	$52,269
Other	15,231,785	$64,317,973,204	$113,654,085,383	$712,228,646,085	$890,200,704,672	$58,444
Private/Commercial/PPO/HMO	2,776,540	$12,348,412,934	$23,823,408,746	$137,372,720,655	$173,544,542,335	$62,504
Rural/Urban						
Urban	24,549,308	$134,880,605,547	$179,608,748,796	$1,164,443,500,000	$1,478,932,854,343	$60,243
Rural	6,292,866	$31,930,196,077	$41,617,835,131	$286,073,751,174	$359,621,782,382	$57,148
Disposition						
Treated and Released	28,387,504	$66,949,530,984	$86,132,322,982	$788,923,246,256	$942,005,100,222	$33,184
Admitted	2,650,568	$101,018,722,894	$136,983,778,930	$672,559,644,444	$910,562,146,268	$343,535
Injury Severity Score (ISS)						
0	4,007,518	$23,294,466,781	$9,224,703,223	$78,422,515,363	$110,941,685,367	$27,683
< 5	23,472,855	$81,231,767,072	$125,971,348,386	$797,810,907,004	$1,005,014,022,462	$42,816
5–14	1,533,316	$31,204,880,868	$36,049,311,657	$287,923,572,318	$355,177,764,843	$231,640
> 15	2,024,383	$32,237,139,158	$51,870,738,645	$297,325,896,016	$381,433,773,819	$188,420
Region						
Northeast	486,635	$18,095,855,894	$24,090,562,684	$119,160,902,927	$161,347,321,505	$331,557
Midwest	601,215	$21,715,142,226	$29,756,502,527	$144,768,266,911	$196,239,911,664	$326,406
South	1,027,654	$36,344,852,910	$52,530,343,991	$258,276,688,480	$347,151,885,381	$337,810
West	535,064	$24,862,871,864	$30,606,369,728	$150,353,786,126	$205,823,027,718	$384,670
Hospital Type						
Nonteaching	11,451,866	$26,985,059,893	$34,347,299,866	$311,700,719,574	$373,033,079,333	$32,574
Teaching	11,244,316	$26,914,053,295	$35,054,247,432	$316,475,541,375	$378,443,842,102	$33,656

### Costs

Hospital-treated nonfatal injuries in 2013 cost an estimated $1.853 trillion, including $168 billion in medical spending, $223 billion in work losses, and quality of life losses valued at $1.461 trillion (Table 1). The total estimated cost per injury was approximately $59,700, including approximately $5400 in medical spending, $7200 in lost future work and $47,100 in quality of life losses. The total costs per injury were highest for the oldest and youngest age groups; individuals < 1 year old ($97,623) and 65 years and older ($71,493). Total cost per injury was slightly higher for those with Medicare and Medicaid versus those with commercial insurance or other payer types. While 91.5% of patients with injuries were discharged, this represented only 8.8% of costs. In contrast, the 8.5% of patients admitted represented 91.2% of costs.

Falls and struck by/against injuries contributed to 35% of nonfatal injury costs (Table 2) and were the leading causes in all age groups (results not shown). The most severe and debilitating injuries will result in higher costs. Among hospital-treated nonfatal injuries, near-drownings, self-harm, and firearm-related violence are the most costly. The external cause of injury was not coded for cases accounting for 9% of total injury costs.

**Table 2:** Cost of Hospital-Treated Nonfatal Injuries in the United States by Mechanism, 2013 (2013 United States $)

	Number	Medical	Work Loss	Quality of Life	Total	Total Cost per Injury
UNINTENTIONAL INJURIES						
Cut/pierce	1,912,149	$3,631,594,141	$4,922,277,919	$28,292,232,329	$36,846,104,389	$19,269
Drowning/submersion	9762	$167,756,380	$585,909,962	$2,699,855,258	$3,453,521,600	$353,773
Fall	8,810,752	$64,201,479,065	$72,918,304,289	$505,230,956,245	$642,350,739,599	$72,905
Fire/flame	80,131	$754,650,203	$728,172,630	$5,538,602,465	$7,021,425,298	$87,625
Hot object/substance	292,856	$988,725,136	$1,708,949,240	$12,359,395,079	$15,057,069,455	$51,415
Firearm	35,858	$358,159,858	$703,471,847	$2,983,640,338	$4,045,272,043	$112,815
Machinery	113,545	$544,119,276	$1,846,062,780	$10,644,611,515	$13,034,793,571	$114,798
MVT Occupant	2,498,200	$13,763,057,426	$22,257,235,570	$97,781,307,804	$133,801,600,800	$53,559
MVT Motorcyclist	157,995	$2,736,135,308	$5,580,436,675	$21,245,276,186	$29,561,848,169	$187,106
MVT Pedal cyclist	53,983	$527,900,384	$1,123,476,662	$5,165,720,968	$6,817,098,014	$126,281
MVT Pedestrian	137,296	$2,181,788,505	$3,548,142,901	$16,715,035,234	$22,444,966,640	$163,478
MVT Unspecified	219,970	$1,053,152,143	$1,820,906,676	$9,972,036,013	$12,846,094,832	$58,399
MVT Other	17,891	$111,877,667	$248,974,328	$1,464,181,060	$1,825,033,055	$102,010
Pedal cyclist, other	324,071	$1,476,820,932	$3,263,738,075	$20,108,440,875	$24,848,999,882	$76,678
Pedestrian, other	19,026	$169,750,289	$289,186,145	$1,884,173,134	$2,343,109,568	$123,153
Transport, other	294,317	$2,358,186,236	$5,066,645,020	$29,023,314,277	$36,448,145,533	$123,840
Bites and stings	1,234,452	$2,547,309,358	$2,869,717,927	$27,803,209,134	$33,220,236,419	$26,911
Other natural/environmental	178,925	$1,168,724,588	$1,414,612,181	$6,270,658,638	$8,853,995,407	$49,484
Overexertion	2,335,656	$6,555,552,120	$10,283,916,364	$65,455,667,267	$82,295,135,751	$35,234
Poisoning	469,441	$2,957,904,882	$836,596,717	$19,797,098,590	$23,591,600,189	$50,255
Struck by/against	3,274,620	$8,646,126,741	$14,196,566,363	$142,802,749,122	$165,645,442,226	$50,585
Suffocation	37,714	$2,035,124,771	$572,859,762	$2,216,460,767	$4,824,445,300	$127,921
Other spec & classification	1,108,881	$4,457,686,594	$4,606,013,305	$54,204,054,262	$63,267,754,161	$57,055
Other specified, NEC	679,383	$2,473,750,166	$3,703,018,649	$23,312,983,807	$29,489,752,622	$43,407
Unspecified	2,116,751	$9,391,792,223	$12,521,017,713	$60,497,266,532	$82,410,076,468	$38,932
SELF-HARM INJURIES						
Cut/pierce	104,661	$555,649,912	$1,526,402,750	$5,323,753,810	$7,405,806,472	$70,760
Drowning/submersion	265	$3,418,777	$21,334,930	$76,901,789	$101,655,496	$383,493
Fall	2377	$133,419,753	$216,937,041	$674,562,123	$1,024,918,917	$431,271
Fire/flame	1561	$47,016,669	$35,395,270	$354,039,886	$436,451,826	$279,685
Hot object/substance	178	$1,007,084	$2,947,776	$35,191,197	$39,146,057	$220,435
Firearm	2686	$192,070,049	$336,463,687	$1,240,360,400	$1,768,894,136	$658,446
MVT Other	823	$19,532,106	$42,591,826	$153,183,991	$215,307,923	$261,588
Other natural/environmental	123	$2,690,121	$4,270,614	$13,651,834	$20,612,569	$167,359
Poisoning	279,444	$2,061,419,009	$799,796,068	$9,522,661,850	$12,383,876,927	$44,316
Suffocation	9076	$123,411,128	$873,402,854	$2,603,923,204	$3,600,737,186	$396,752
Other specified & classifiable	1875	$73,774,938	$172,400,608	$544,485,818	$790,661,364	$421,619
Other specified, NEC	27,623	$175,461,682	$411,227,781	$1,413,948,899	$2,000,638,362	$72,426
Unspecified	7271	$34,820,468	$79,108,294	$270,489,268	$384,418,030	$52,869
ASSAULT						
Cut/pierce	73,932	$491,701,003	$1,138,106,551	$5,476,377,890	$7,106,185,444	$96,118
Drowning/submersion	69	$642,802	$1,964,335	$7,164,047	$9,771,184	$141,881
Fall	1646	$12,022,160	$37,569,063	$169,630,025	$219,221,249	$133,196
Fire/flame	595	$3,858,466	$9,261,217	$69,978,211	$83,097,894	$139,678
Hot object/substance	1962	$15,993,005	$17,535,750	$153,556,467	$187,085,222	$95,368
Firearm	30,131	$927,098,739	$1,530,254,784	$6,968,840,319	$9,426,193,842	$312,844
MVT Occupant	1660	$6,798,022	$12,494,180	$130,515,813	$149,808,015	$90,225
Poisoning	1761	$7,901,485	$2,498,250	$92,294,308	$102,694,043	$58,317
Struck by/against	611,218	$2,512,923,350	$6,795,903,883	$56,416,139,291	$65,724,966,524	$107,531
Suffocation	3006	$10,208,810	$27,416,530	$127,393,050	$165,018,390	$54,888
Other specified & classifiable	119,372	$604,801,671	$1,005,672,954	$7,496,925,042	$9,107,399,667	$76,294
Other specified, NEC	165,835	$571,842,316	$1,014,664,619	$11,060,805,689	$12,647,312,624	$76,265
Unspecified	169,769	$879,584,274	$2,260,749,692	$17,139,072,436	$20,279,406,402	$119,453
UNDETERMINED INTENT						
Cut/pierce	3731	$13,136,329	$31,468,294	$124,000,521	$168,605,144	$45,196
Drowning/submersion	233	$912,250	$8,301,876	$31,576,211	$40,790,337	$175,358
Fall	3950	$39,802,960	$99,196,652	$333,040,805	$472,040,417	$119,510
Fire/flame	2913	$15,518,883	$31,234,477	$242,655,585	$289,408,944	$99,345
Hot object/substance	595	$3,699,041	$7,221,322	$73,345,138	$84,265,501	$141,740
Firearm	4014	$74,095,787	$129,846,857	$546,583,710	$750,526,354	$186,991
MVT Other	459	$3,147,206	$6,780,905	$29,228,290	$39,156,401	$85,342
Other natural/environmental	2703	$58,901,837	$45,949,242	$145,873,538	$250,724,617	$92,761
Poisoning	126,707	$892,799,765	$326,704,590	$5,843,044,484	$7,062,548,839	$55,739
Suffocation	267	$1,684,991	$9,729,591	$19,947,053	$31,361,635	$117,601
Other specified & classifiable	463	$4,000,582	$9,817,663	$43,121,482	$56,939,727	$122,885
Other specified, NEC	14,025	$64,785,622	$98,439,086	$539,613,549	$702,838,257	$50,114
Unspecified	76,146	$313,398,577	$320,997,827	$2,566,738,535	$3,201,134,939	$42,039
LEGAL INTERVENTION^a^						
Cut/pierce	1449	$3,102,948	$6,635,734	$22,866,321	$32,605,003	$22,502
Firearm	1383	$41,617,599	$57,218,311	$234,236,902	$333,072,812	$240,832
Poisoning	349	$645,957	$336,617	$13,470,021	$14,452,595	$41,469
Struck by/against	41,824	$127,374,328	$266,063,345	$1,325,708,220	$1,719,145,893	$41,104
Other specified & classifiable	1174	$9,406,164	$8,221,940	$41,502,028	$59,130,132	$50,375
Other specified, NEC	121	$1,064,568	$2,915,549	$9,994,667	$13,974,784	$115,142
Unspecified	6937	$20,238,437	$42,810,948	$214,315,994	$277,365,380	$39,984
UNSPECIFIED INTENT						
Drowning/submersion	443	$27,326,952	$54,731,485	$204,710,316	$286,768,754	$646,970
Other natural/environmental	49,694	$686,083,672	$522,349,852	$2,757,831,073	$3,966,264,597	$79,814
Poisoning	81,013	$677,091,885	$163,997,422	$4,002,093,054	$4,843,182,361	$59,783
Suffocation	28,327	$1,760,326,274	$379,312,268	$1,302,485,774	$3,442,124,316	$121,514
Other specified & classifiable	57,020	$351,856,081	$246,844,436	$698,824,829	$1,297,525,346	$22,756
Unspecified	2,499,592	$18,048,042,991	$24,246,394,615	$139,111,309,043	$181,405,746,649	$72,574

*MVT* Motor vehicle traffic

*NEC* Not elsewhere classifiable

*Deaths due to injuries inflicted by police or other law enforcement agents

## Discussion

Approximately one in 10 individuals in the United States is injured each year are treated in a hospital, with high corresponding costs. Our calculated rate of 9.8 injuries per 100 people in 2013 is nearly half that of the 18.135 injuries per 100 population estimate by Corso et al. based on data from 2000. (Corso et al. 2006) While a prior study of US spending on personal health care found that injuries accounted for $168 billion (8%) of the $2.1 trillion in medical spending in 2013, our comprehensive injury medical spending estimate exceeds that, even while excluding the 12 million injuries treated in physician’s offices and clinics. (Dieleman et al. 2016) Limitations of the study include the absence of outpatient and ambulatory data, as well as the possibility of misclassification based on ICD codes.

These cost data from 2013 support priority-setting and selection of interventions to reduce the burden of injury in the United States.

## Abbreviations

ED: Emergency department
HCUP: Healthcare Cost and Utilization Project
MVT: Motor vehicle traffic
NEC: Not otherwise classifiable
